# Macroscopic fracture mechanism of coal body and evolution characteristics analysis of impact force in deep coal and gas outburst

**DOI:** 10.1038/s41598-023-43100-2

**Published:** 2023-09-24

**Authors:** Lingran Ren, Jupeng Tang, Yishan Pan, Xin Zhang, Honghao Yu

**Affiliations:** 1 School of Mechanics and Engineering, Liaoning Technical University, Fuxin, 123000 Liaoning People’s Republic of China; 2 School of Physics, Liaoning University, Shenyang, 110036 Liaoning People’s Republic of China

**Keywords:** Fossil fuels, Geology, Mineralogy

## Abstract

With the increase of mining depth and intensity, coal and gas outburst dynamic disasters occur frequently. In order to deeply study the macroscopic fracture mechanism of coal body and evolution characteristics analysis of impact force, taking the outburst coal seam of Pingmei No. 11 Coal Mine and Sunjiawan coal seam of Hengda Coal Mine as the research objects, the simulation roadway test system of self-developed true triaxial coal and gas outburst is applied to carry out the simulation test of deep coal and gas outburst with buried depths of 1000 m, 1200 m, 1400 m and 1600 m. During the test, the overlying strata stress is simulated by axial compression, the surrounding rock stress is simulated by confining pressure, the gas pressure is simulated by pore pressure, the impact force and acoustic emission monitoring technology are introduced, and the coal seam gas pressure is simulated by mixture pressure of 45% CO_2_ and 55% N_2_. From the viewpoint of fracture mechanics, the crack propagation mechanism of coal in the outburst launching area is discussed, the evolution characteristics of impact force and gas pressure are analyzed, and the influence law between acoustic emission signal and impact force is revealed. From the viewpoint of energy conversion, the transformation character of gas internal energy to impact kinetic energy (gas pressure to impact force) are analyzed. The results show that the generation of I-type crack is a prerequisite for outburst catastrophe. With the crack propagation, I-type and II-type cracks intersect and penetrate, resulting in internal structural damage and skeleton instability of coal. Gas wrapped fragmentized coal body thrown, outburst occurs. There is obvious negative pressure in the roadway after outburst. The occurrence of negative pressure is greatly affected by the physical and mechanical properties of coal, ground stress and gas pressure. Impact kinetic energy is mainly provided by gas internal energy. Part of the gas pressure is converted into impact force. The strength and duration of the impact force are determined by the gas pressure. Under the condition of deep working conditions (high ground stress and low gas pressure), the propagation of impact force in the roadway is more hindered. Both impact force and acoustic emission signals can monitor the occurrence of outburst. The peak point of acoustic emission ringing count is earlier than the impact force. The acoustic emission signal can monitor the outburst hazard earlier. The impact force can more specifically reflect the coal fracture.

## Introduction

Energy is an important material basis for the survival and development of human society. According to the energy structure characteristics of China's “gas starvation, less oil and relatively rich coal”, China is highly dependent on coal consumption^1^. The Chinese Academy of Engineering predicts that the proportion of coal in primary energy consumption will remain at about 50% in 2050, and coal will the dominant energy in China in the coming 30 years^2,3^. However, with the gradual depletion of shallow resources, deep mining has become a necessary trend. At present, the maximum mining depth of deep mines in China has reached 1501 m. The mining depth is still increasing year by year, with an increasing speed of 10–25 m every year. In the coming 5–10 years, more than 30 deep mines below 1000 m will be built^4–6^. Among them, deep mines are mainly concentrated in Northeast China, East China, Central China and North China^7^. With the construction of deep mines, some low gas coal mines have a tendency to change to high outburst coal mines. Under the disturbance of deep mining, the probability of mine dynamic disasters increase. Among them, coal and gas outburst (abbreviation as outburst) disasters are the most frequent and harmful^8^.

Outburst is a mechanical process caused by the sudden failure of coal under the coupling effect of coal deformation and gas flowing^9^. The deep does not refer to the specific depth, but a mechanical state determined by the ground stress level, mining stress state and surrounding rock property^5^. Since the outburst of Issac coal mine in Loire coalfield in France was recorded in 1834, researchers had done a lot of researches on the occurrence mechanism, phenomenon and prevention measures of outburst. So far, there is no unified and complete theoretical system for the outburst mechanism. The four generally acknowledged types of outburst mechanism hypotheses are gas leading role hypothesis^10,11^, ground stress leading role hypothesis^12–14^, nature of chemistry role hypothesis^15^, and comprehensive role hypothesis^16–19^. Among them, the comprehensive role hypothesis (that outburst is the result of the combined action of ground stress, gas pressure and physical mechanical properties of coal) is widely accepted. With the further research of the outburst mechanism and outburst prevention technology, the incidence of outburst disasters has been significantly reduced, but it still cannot be completely exclude. Therefore, further research on the disaster-causing mechanism of outburst is still a difficult problem to be overcome. Considering the strong destructibility, uncertainty, intensity and other harmful factors of coal mine site outburst, scholars mainly study through theoretical analysis, physical similarity simulation test and numerical simulation.

**Figure 1:**
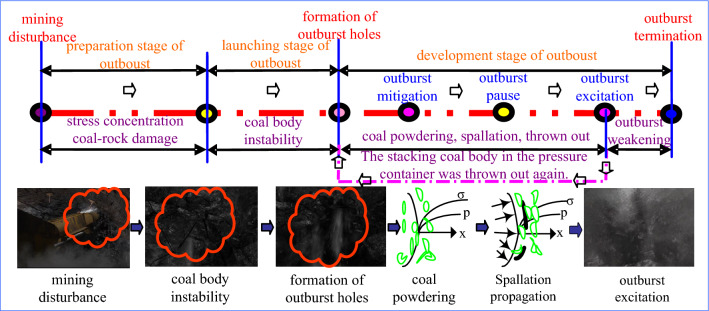
Coal and gas outburst process description.

The existing research has shown the essence of outburst catastrophe is that mining disturbance and ground stress lead to the initial failure of coal-rock, which becomes the premise of disaster. At this time, gas adsorption and desorption begin, which changes the mechanical response characteristics of gassy coal-rock, weakens the mechanical strength of coal-rock, reduces the ability to resist external load, and promotes the occurrence of structural damage deformation and Fracture instability phenomenon of coal-rock^20–24^. This shows the characteristics of multi-source composite co-occurrence of outburst disasters. In the face of the serious situation of outburst disaster-prone, it is urgent to study the mechanism and process of “germinate disaster-lead to disaster” (Fig. 1).

At present, some scholars focus on the study of outburst impact force and shock wave. The propagation law of shock wave and impact airflow in roadway is monitored by self-developed outburst equipment^25–27^. The single fluid of outburst is extended to coal–gas two-phase flow, and the migration law of coal–gas two-phase flow is emphatically analyzed^28–32^. Pulverized coal flow is mainly reflected in the subsidence law of pulverized coal^33,34^, and the impact airflow is related to the propagation of impact force and shock wave^35,36^.

In summary, the research on the essence of outburst catastrophe, the evolution law of impact force and the disaster-causing mechanism of impact have become the main direction of current research. At present, scholars from China and other countries take shock wave and impact airflow as the research object, and rarely consider the impact force evolution of the same roadway monitoring point of different coal samples at different times, or only consider the influence of gas pressure on impact force, without considering the conversion relationship between gas pressure and impact force. The authors used the simulation roadway test system of self-developed true triaxial coal and gas outburst to carry out the simulation test of deep coal and gas outburst with different buried depths. From the viewpoint of fracture mechanics, the crack propagation mechanism of coal in the outburst launching area is discussed, the evolution law of impact force at the same roadway monitoring point, different coal samples and different time are analyzed. The relationship between gas pressure and impact force is obtained from the viewpoint of outburst energy conversion, and the relationship between impact force and acoustic emission signal is studied. The aim is to provide reference for the essence of deep outburst disaster and the disaster-causing mechanism of impact.

## Experimental design of deep coal and gas outburst

There are significant difference between deep mining environment, gas existing conditions and shallow. Outburst field observation has strong risk. The laboratory test has become an important means to study the outburst laws. The simulation roadway test system of self-developed true triaxial coal and gas outburst is studied. The ground stress environment of coal body is simulated by triaxial stress loading. The coal seam gas pressure is simulated by mixture pressure of 45% CO_2_ and 55% N_2_. In the meantime, the parameter variation of acoustic emission signals and impact force in the outburst process are monitored in real time. It is helpful for further study of deep outburst.

### Experimental coal samples

The experimental coal samples were taken from Pingmei No. 11 Coal Mine of Pingdingshan High Gas Mine in Henan Province and Sunjiawan Coal Mine of Fuxin Hengda Coal Mine in Liaoning Province. Domestic deep mines are mainly concentrated in Northeast, East and Central China as well as North China. The No. 11 Coal Mine and Hengda Coal Mine are located in central China and northeast China respectively, both of which are mainly concentrated in deep mines. Pingdingshan coal mine has abundant reserves, excellent coal quality, stable coal seams, simple structure, and less complicated mining technical conditions. In the northeast region, the mining geological conditions are complex, and the threat of coal mine disasters is serious. Using coal samples from coal mines with different geological conditions to conduct coal and gas outburst tests can mutually verify the universality of the conclusions obtained from the tests. In the No. 11 Coal Mine, the original gas pressure is 0.21–0.67 MPa, the gas content is 2.28–5.65 m^3^/t, the absolute gas emission quantity is 0.37–0.71 m^3^/min, and the relative gas emission quantity is 2.79–3.34 m^3^/t. According to geological exploration data and coal seam exposure condition, the average total thickness of coal seam is about 5.34 m, the maximum average thickness is about 1.82 m, and the density is approximately 1.29 t/m^3^. The high gas coal seam of Sunjiawan in Hengda Coal Mine belongs to the coal-bearing stratum in Liujia District, Fuxin^37^. The gas content of the coal seam is 7–10 m^3^/t, the average thickness is about 1.2 m, the apparent density is 1.52 t/m^3^, the true density is 1.593 t/m^3^, and the compressive strength is 14.42–31.28 MPa.

### Test system

**Figure 2:**
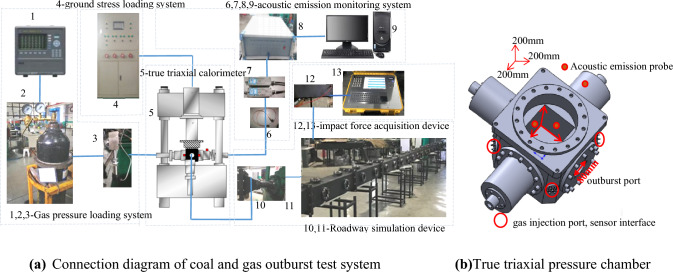
Diagrammatic drawing of coal and gas outburst test system.

The test adopts the simulation roadway test system of self-developed true triaxial coal and gas outburst, as shown in Fig. 2.

The size of the true triaxial pressure chamber is 200 mm × 200 mm × 200 mm. The left and right sides, the bottom and the rear are equipped with hydraulic cylinders to provide triaxial stress, up to 25 MPa. An outburst weak plane with diameter of 80 mm was installed at the front-end of outburst port to induce the outburst to occur. There are 4 gas injection ports and 24 sensor interfaces around. Considering the danger of gas, according to the concept of methane similar gas, the adsorption strength of coal is CO_2_ > CH_4_ > N_2_ for the gases commonly used in previous tests. To ensure that the adsorption is consistent with methane, a mixture of CO_2_ and N_2_ is most appropriate. It was found that the outburst phenomenon and the outburst critical value of the gas mixture with volume fractions of 45% CO_2_ and 55% N_2_ were similar to those of gas through experimental study. The mixture pressure of 45% CO_2_ and 55% N_2_ is used to simulate the gas pressure of coal seam^38^. Combined with the pressure resistance and economy, the roadway simulation device is composed of four straight roadways with a length of 1000 mm and a thickness of 12 mm, 10 mm and 8 mm. The outburst surface is connected with the roadway through the flange plate, transition section and expanding section. A sensor installation hole is set on the right side of the roadway closest to the outburst port to monitor the propagation law of impact force. The impact force is collected by a manually triggered voltage-type overpressure sensor. The collected data is directly displayed on the impact tester panel, which can quickly and intuitively monitor the changes of impact force. DS5 series full information acoustic emission monitoring device can extract the acoustic emission signals during the test in real time, through the acoustic and electrical signal conversion, and finally collected in the form of digital signals, which can be used to interpret the rupture of coal and rock. The amplifier gain is 40 dB, and the threshold value is set to 52–65 mv according to the test before each test in order to eliminate the interference of ambient noise to the test data.

### Test scheme and steps

Most scholars believe that the "deep" of Chinese deep mining can be defined as 800–1500 m. Among them, 700–1000 m is the general deep part, and 1000–1200 m is the super deep part. The gas outburst quantity at a depth of 600–900 m increased significantly.Combined with the “deep” scheme and gas outburst quantity in deep mining in China, the buried depth of 600–800 m is considered as the upper of deep mine^39–42^. Therefore, the simulated burial depth range is 1000–1600 m. Considering that the ground stress measurement of deep coal-rock is difficult, the fitting formula of ground stress calculation obtained by Li Xinping^43^ is applied to determine the ground stress value of coal-rock under different work conditions. In order to ensure the safety of laboratory loading, similarity equation of mechanics were utilized:

1 $$C_{1} = \frac{{C_{\sigma } }}{{C_{L} C_{\rho } }} = 1$$

where $C_{\sigma }$ is the stress similarity coefficient, $C_{L}$ is the geometric similarity constant, $C_{L} = {{L_{p} } \mathord{\left/ {\vphantom {{L_{p} } {L_{m} }}} \right. \kern-0pt} {L_{m} }}$, $L_{p} = 1.82\;{\text{m}}$, $L_{m} = 0.2\;{\text{m}}$, $C_{\rho }$ is the volume force similarity constant, $C_{L} = {{\rho_{p} } \mathord{\left/ {\vphantom {{\rho_{p} } {\rho_{m} }}} \right. \kern-0pt} {\rho_{m} }}$, $\rho_{p} = 1.29\;\left( {{\text{t}} \cdot {\text{m}}^{{ - 3}} } \right)$, $\rho_{m} = 1.088\;\left( {{\text{t}} \cdot {\text{m}}^{{ - 3}} } \right)$, $p$ represents raw coal, $m$ represents briquette. The stress similarity ratios of Sunjiawan coal seam in Pingdingshan No. 11 Coal Mine and Hengda Coal Mine are 10.8 and 8.3 respectively, and the gas pressure similarity ratio is 1.0. Then, the loading values of vertical ground stress, maximum horizontal ground stress, minimum horizontal ground stress and gas pressure are obtained. In order to achieve the jet effect during the test, according to the “Coal Mine Safety Production Regulations”, the initial value of gas pressure loading is set to 0.6 MPa. The detail loading scheme is shown in Table 1.

**Table 1:** Loading scheme of coal and gas outburst.

Coal mine	Pingmei No. 11 Coal Mine			Sunjiawan coal seam of Hengda coal mine			Gas pressure (MPa)
Simulated buried depth (m)	Test ground stress (MPa)						
	*σ*_*H*_*′*	*σ*_*h*_*′*	*σ*_*v*_*′*	*σ*_*H*_*′*	*σ*_*h*_*′*	*σ*_*v*_*′*	
1000	2.91	1.79	2.13	3.80	2.30	2.70	Vacuum for 3 h, when the negative pressure in the cavity is 0.1 MPa, the gas is filled to simulate adsorption for 24 h. The initial loading stress of the test was 0.6 MPa, the load per stage is increased by 0.2 MPa, and the pressure was stabilized for 2 min each time until the outburst occurred
1200	3.35	2.13	2.51	4.36	2.77	3.27	
1400	3.79	2.47	2.90	4.93	3.21	3.77	
1600	4.23	2.81	3.28	5.51	3.66	4.27	

**Figure 3:**
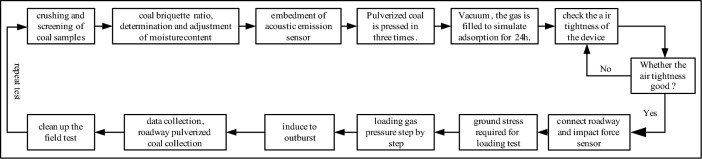
The specific test steps of coal and gas outburst.

The specific test steps are shown in Fig. 3.

## Experimental phenomena and failure mechanism

### Experimental phenomena

**Figure 4:**
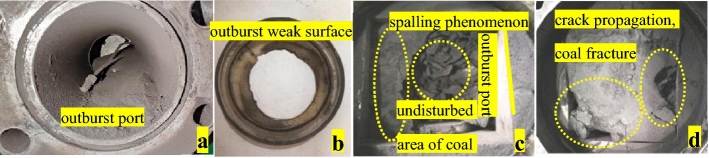
The phenomenon of coal and gas outburst.

Outburst is a phenomenon of dynamic instability. Under different buried depth conditions, it is affected by ground stress and gas pressure differently. When the gas pressure disturbance reaches the critical value, the outburst weak surface ruptures. The gas gathered near the outburst mouth is wrapped in the broken coal sample and sprayed out. The gas gathered near the outburst port wrapped fragmentized coal body thrown. It has the characteristics of great intensity, strong destructibility and rapid speed. The rupture area of the outburst weak surface is more than 90% (Fig. 4a,b). Under the disturbance of gas, the coal samples in the pressure chamber are divided into the undisturbed area of coal seam and the outburst launching area. The coal samples in the launching area undergo the process of “crack formation-crack propagation-breakup” (Fig. 4d). After the outburst is completed, the remaining coal samples in the launching area show obvious “spalling phenomenon” (Fig. 4c). The experimental results are in agreement with previous research^44^, indicating the reliability of the test.

**Figure 5:**
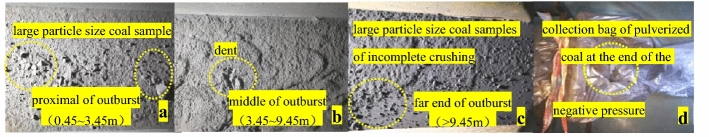
The test phenomenon in the simulated roadway.

The outburst pulverized coal is mainly concentrated in the middle and proximal of the roadway, mainly small particle size coal samples (Fig. 5a,b). Among them, there is a visual large-sized coal sample at the proximal of outburst (Fig. 5a). The small particle size coal samples are affected by the two-phase flow impact force and air resistance, and the subsidence decreases gradually with the increase of the outburst distance. The large particle size coal samples are crushed and subsided under the action of impact force. The sedimentary part of incomplete crushing causes a dent in the middle of the outburst during the migration process (Fig. 5b). With the increase of outburst duration, it is migrated to the far end of outburst (the end of roadway, Fig. 5c). It shows that the subsidence of pulverized coal has obvious selective characteristics^45^, which is consistent with the actual underground outburst. The collection bag of pulverized coal at the end of the roadway is sucked into the roadway (Fig. 5d). The reason is that the velocity of coal–gas two-phase flow is fast, and the air in the simulated roadway is compressed, so that the interface between two-phase flow and air is squeezed. It is confirmed that there is negative pressure in the roadway during the outburst process, which is similar to the previous experimental phenomenon^46^.

### Crack propagation mechanism of coal body in the outburst launching area

After loading the ground stress according to the test scheme, it remains constant, and the gas pressure increases step by step until the outburst occurs, which is consistent with the existing research on the loading of catastrophic stress^47,48^. The initial failure of coal containing gas caused by ground stress loading and the generation of large newly-generated cracks are the premise of catastrophe. With the injection of gas, the mechanical strength of coal body and the ability to resist external load are reduced, which promotes the further extension of cracks. Coal body fracture deformation, instability and failure, induced outburst. The lunch of outburst indicates that some coal containing gas have experienced local damage, extensive failure, overall instability and throwing stage. Therefore, there are four periods for any particle failure of coal body in the outburst lunching area: initial failure period of ground stress, Stage of crack propagation by gas, outburst period of coal body instability, period of transportation and stop^49^.

**Figure 6:**
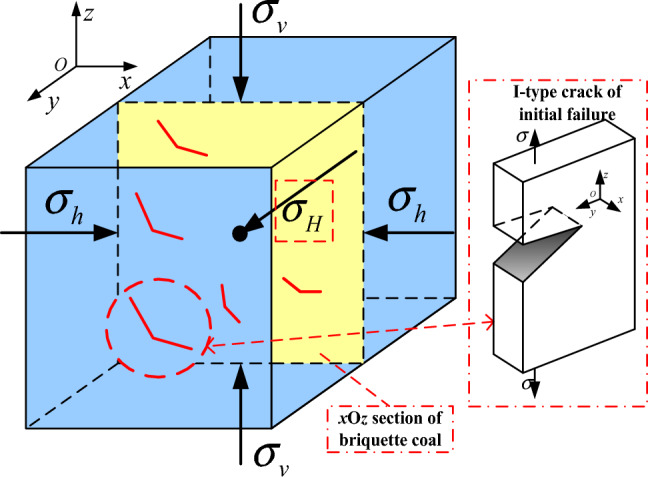
Force analysis of initial failure of coal containing gas.

According to the fracture characteristics of the specimen, the cracks can be divided into opening type crack (I-type), sliding mode crack (II-type) and tearing mode crack (III-type). Considering the initial force condition of coal body in the pressure chamber after only loading ground stress, the specimen only produces I-type cracks, as shown in Fig. 6. The any section *x*O*z* is intercepted for analysis. At the initial stage of gas pressure loading, the coal briquette is in an adsorption saturation state. The ground stress loading caused the initial failure of coal containing gas in the launch area, resulting in I-type crack, which became a prerequisite for outburst catastrophe.

**Figure 7:**
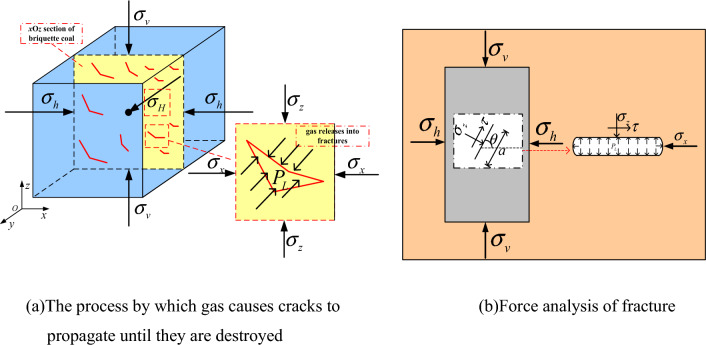
Force analysis of crack propagation of coal containing gas.

The ground stress is stable. As the gas pressure is loaded, the coal body enters the Stage of crack propagation by gas, as shown in Fig. 7a. Under the synergistic effect of ground stress and gas pressure, a large newly-generated cracks are generated in the coal body of the launching area. The adsorbed gas near fracture becomes free and moves inside the fracture. When the gas pressure in the crack reaches the stress value of the crack, the gas pressure has a stretching effect on the crack and accelerates the crack propagation speed^50^. After the crack propagation, the adsorbed gas near crack continues to desorb, repeating the above process. Under the sustained action of gas pressure, the coal structure in the launch area is completely destroyed and the skeleton lose its stability. When the gas pressure increases up to a critical value, the outburst baffle is broken, the broken coal is thrown out, and the outburst occurs.

The any section *x*O*z* in the tiny unit containing cracks in the coal body of the launching area is taken for force analysis. It is assumed that the selected tiny unit has all the characteristics of the coal body in the launch area. The fracture expands and the stress is locally unloaded along the direction of the outburst port. The external of the fracture is squeezed by axial compression and confining pressure. The interior of the fracture is expanded by gas pressure. When the expansion effect of gas pressure is greater than or equal to the extrusion effect of axial compression and confining pressure, the crack extends outward. With the expansion of the pre-existing fracture, the fracture gradually appears dip angle. The original stress will have a shearing effect on the fracture surface, as shown in Fig. 7b. The compressive stress of the specimen is used to represent the stress on the crack surface. The expression is:

2 $$\left\{ \begin{gathered} \tau = {{\left( {\sigma_{v} - \sigma_{h} } \right)\sin 2\theta } \mathord{\left/ {\vphantom {{\left( {\sigma_{v} - \sigma_{h} } \right)\sin 2\theta } 2}} \right. \kern-0pt} 2} \hfill \\ \sigma_{z} = {{\left[ {\left( {\sigma_{v} + \sigma_{h} } \right) + \left( {\sigma_{v} - \sigma_{h} } \right)\cos 2\theta } \right]} \mathord{\left/ {\vphantom {{\left[ {\left( {\sigma_{v} + \sigma_{h} } \right) + \left( {\sigma_{v} - \sigma_{h} } \right)\cos 2\theta } \right]} 2}} \right. \kern-0pt} 2} \hfill \\ \sigma_{x} = {{\left[ {\left( {\sigma_{v} + \sigma_{h} } \right) - \left( {\sigma_{v} - \sigma_{h} } \right)\cos 2\theta } \right]} \mathord{\left/ {\vphantom {{\left[ {\left( {\sigma_{v} + \sigma_{h} } \right) - \left( {\sigma_{v} - \sigma_{h} } \right)\cos 2\theta } \right]} 2}} \right. \kern-0pt} 2} \hfill \\ \end{gathered} \right.$$

where $\tau$ is the shear stress on the crack surface, MPa. $\sigma_{z}$ is the normal stress on the crack surface, MPa. $\sigma_{x}$ is the transversal normal stress on the crack surface, MPa. $\theta$ is the inclination of primary crack, °. $\sigma_{v}$ and $\sigma_{h}$ are the compressive stress of the specimen (pressure is positive), MPa.

The transversal normal stress is parallel to the cracking direction. The thickness and tip radius of curvature of the I-crack are non-zero value. Therefore, the stress intensity factor generated by transversal normal stress is:

3 $$K_{I\left( x \right)} = Y\left( {\left| {P_{L} } \right| - \sigma_{x} } \right)\sqrt {{\rho \mathord{\left/ {\vphantom {\rho a}} \right. \kern-0pt} a}} \sqrt {{{\pi a} \mathord{\left/ {\vphantom {{\pi a} 2}} \right. \kern-0pt} 2}}$$

where $K_{I}$ is the stress intensity factor, which is a parameter to measure the stress field intensity, MPa∙m^0.5^. $Y$ is the coefficient of crack shape, the dimension is 1, which is related to the geometry and loading mode of crack^51^. $P_{L}$ is the gas pressure in the fracture, MPa. $a$ is the crack length, m. The establish condition of Eq. (3) is that $\frac{\rho }{a}$ approaches 0, so the $K_{{{\rm I}(x)}}$ is ignored.

Therefore, the stress intensity factor of I-crack is:

4 $$K_{I} = Y\left( {\left| {P_{L} } \right| - \sigma_{z} } \right)\sqrt {{{\pi a} \mathord{\left/ {\vphantom {{\pi a} 2}} \right. \kern-0pt} 2}}$$

According to the fracture mechanics theory, the conditions of crack propagation is:

5 $$K_{I} = K_{Ic}$$

where $K_{Ic}$ is the fracture toughness of coal body, which is the capability index of coal body to prevent crack propagation and fracture. It is the inherent attribute of coal body^52^, MPa∙m^0.5^.

When $Y\sigma_{z} \sqrt {\frac{\pi a}{2}} = K_{Ic}$, the conditions of crack propagation is satisfied. Substituting Eqs. (2) and (4) into Eq. (5), the I-crack propagation criterion of coal body in the launching area under gas pressure is:

6 $$\left| {P_{L} } \right| - {{\left[ {\left( {\sigma_{v} + \sigma_{h} } \right) + \left( {\sigma_{v} - \sigma_{h} } \right)\cos 2\theta } \right]} \mathord{\left/ {\vphantom {{\left[ {\left( {\sigma_{v} + \sigma_{h} } \right) + \left( {\sigma_{v} - \sigma_{h} } \right)\cos 2\theta } \right]} 2}} \right. \kern-0pt} 2} \ge {{\sqrt 2 K_{Ic} } \mathord{\left/ {\vphantom {{\sqrt 2 K_{Ic} } {Y\sqrt {\pi a} }}} \right. \kern-0pt} {Y\sqrt {\pi a} }}$$

When the gas pressure in the fracture satisfies this condition, the fracture expands and lengthens. With the expansion of the fracture, the gas concentration in the fracture decreases. The adsorbed gas in the coal near the fracture moves rapidly to the fracture, which makes the gas pressure in the fracture rise again and repeats the above process many times.

With the continuous desorption and filling of gas, the tensile expansion of crack increases continuously, the inclination of the primary crack increases, and the shear stress on the crack surface is gradually obvious. The crack propagates under shear stress and II-type crack is formed. The stress intensity factor of II-crack is:

7 $$K_{{{\rm I}{\rm I}}} = \tau_{e} \sqrt {{{\pi a} \mathord{\left/ {\vphantom {{\pi a} 2}} \right. \kern-0pt} 2}}$$

where $\tau_{e}$ is the equivalent stress on the crack surface, MPa, $\tau_{e} = \tau + \mu \sigma_{z}$. $\tau$ provides the force for sliding failure, MPa. $\mu$ is the friction coefficient of the crack surface, and the normal stress $\sigma_{z}$ produces frictional resistance against the sliding of the crack surface. Because the initial failure of the coal body in the launch area first produces the I-crack, the frictional resistance is 0, so $\tau_{e} = \tau$.

Therefore, the stress intensity factor of II-crack is:

8 $$K_{{{\rm I}{\rm I}}} = \tau \sqrt {{{\pi a} \mathord{\left/ {\vphantom {{\pi a} 2}} \right. \kern-0pt} 2}}$$

The expansion condition of II-crack is:

9 $$K_{{{\rm I}{\rm I}}} = K_{{{\rm I}{\rm I}c}}$$

When $\tau \sqrt {\frac{\pi a}{2}} = K_{{{\rm I}{\rm I}c}}$, the conditions of crack propagation are satisfied. Substituting Eqs. (2) and (8) into Eq. (9), the II-crack propagation criterion of coal body in the launching area under gas pressure is:

10 $$\left( {\sigma_{v} - \sigma_{h} } \right)\sin 2\theta \ge {{2K_{{{\rm I}{\rm I}c}} } \mathord{\left/ {\vphantom {{2K_{{{\rm I}{\rm I}c}} } {\sqrt {\pi a} }}} \right. \kern-0pt} {\sqrt {\pi a} }}$$

For I-crack, when $K_{I} = K_{Ic}$, the crack begins to propagate. In the process of crack propagation, because the coal body near the crack continuously desorbs the gas to migrate into the crack, the gas concentration in the crack remains basically unchanged, and the gas pressure tends to be constant. It can be seen from Eq. (6) that the value on the left side of the equation remains basically unchanged, and the value on the right side gradually decreases with the extension of crack propagation. The coal fracture in the launching area continues to accelerate extension under the action of gas pressure.

For II-crack, when $K_{{{\rm I}{\rm I}}} = K_{{{\rm I}{\rm I}c}}$, the crack propagates under shear stress. It can be seen from Eq. (10) that the shearing sliding fracture of the crack is unrelated to the gas pressure. With the extension of crack propagation, the inclination of crack increases, and the II-crack formed by shear stress becomes increasingly obvious. With the crack propagation, I-type and II-type cracks intersect and penetrate, macro cracks are formed and coal damage is accelerated, resulting in internal structural damage and skeleton instability of coal. Gas wrapped fragmentized coal body thrown, outburst occurs.

## Evolution characteristics analysis of impact force

During the outburst process, the gas wrapped fragmentized pulverized coal thrown to form the impact air flow, which squeezes the air in the roadway. After multiple superposition propagation, the shock wave is finally formed. The impact air flow attenuates through the barrier of the outburst weak surface. The shock wave attenuates with the increase of the outburst distance. This leads to a small impact force value at the distal end of the roadway, which cannot well reflect the coal fracture in the pressure chamber and the release amount of outburst energy. Therefore, the impact force sensor is installed at the side of the roadway 1 m away from the outburst port to monitor the impact force evolution law of the same measure locations at different buried depths during the test.

### Evolution characteristics of impact force at different buried depths

**Figure 8:**
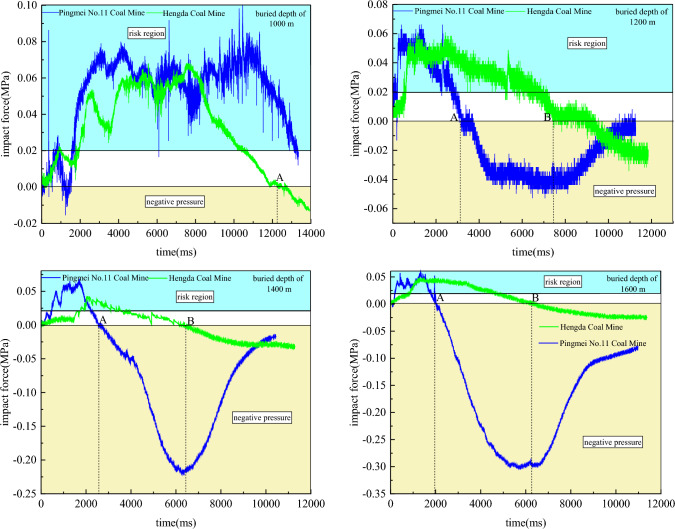
The evolution law of impact force during outburst under different buried depths.

In order to further study the evolution characteristics of impact force, the outburst coal seam of Pingmei No. 11 Coal Mine and Sunjiawan high gas coal seam of Hengda Coal Mine were taken as the research objects, and the impact force of simulated buried depth of 1000 m, 1200 m, 1400 m and 1600 m were monitored (Fig. 8).

The evolution law of impact force at different simulated buried depths of the same measure locations after outburst are as follows:

1. After the impact force rises to the maximum value, the fluctuation intensity is large and the time is long at the simulated buried depth of 1000 m. According to the “deep” scheme, it is considered that the critical depth of the two coal mines can be defined as the simulated buried depth of 1000 m. The buried depth is simulated from 1000 to 1600 m, and the change of impact force follows the law of “fast rise-peak value-slow decrease”. With the increase of simulated buried depth, the ground stress increases gradually, the critical gas pressure required for outburst decreases, and the migration time of broken pulverized coal in roadway is shortened. With the increase of simulated buried depth, the peak value of impact force decreases and the number of peaks decreases. It shows that high ground stress and low gas pressure can hinder the propagation frequency of impact force in roadway under deep work conditions. Therefore, in deep coal mining, the gas extraction should be paid attention to. The purpose of effectively reducing the damage caused by the impact force to the underground mining space and equipment for producing and ensuring the safety of the staff will be achieved.
2. After the outburst, the impact force in the roadway obviously has a negative pressure, which causes the pulverized coal collection bag at the end of the roadway to be sucked into the roadway. The negative pressure increases with the increase of simulated buried depth. When the simulated buried depth reaches a certain value, the suction generated by the negative pressure may gradually be greater than the impact force generated by the outburst. It shows that the staff is more dangerous under the deep work conditions. In addition to the harm caused by the impact force, the staff may also be sucked into the deep part of the roadway and buried. Therefore, with the increase of mining depth and intensity, in order to ensure the safety of underground workers, it has become an inevitable trend to study the mechanism of deep outburst.
3. Point A indicates the occurring time of negative pressure in Pingmei No. 11 Coal Mine, and point B indicates the occurring time of negative pressure in Hengda Coal Mine. When the simulated buried depth is 1000 m, there is no obvious negative pressure in Pingmei No. 11 Coal Mine. The time of negative pressure in Hengda Coal Mine is late, which is 12,303 ms. When the simulated burial depths are 1200 m, 1400 m and 1600 m respectively, the negative pressure time of Pingmei No. 11 Coal Mine is 3160 ms, 2554 ms and 2020 ms respectively, and the negative pressure time of Hengda Coal Mine is 7766 ms, 6411 ms and 6369 ms respectively. With the increase of simulated buried depth, the occurring times of negative pressure in the Pingmei No. 11 Coal Mine and Hengda Coal Mine are advanced, the critical gas pressure decreases, and the propagation time of impact force in roadway becomes shorter. It shows that the strength and duration of impact force are determined by the gas pressure.
4. Comparing the variation of impact force in the two coal mines, it is found that the peak time of impact force is basically the same. The peak value of impact force is not considered. It shows that the variation of impact force in the propagation process of roadway is less affected by the physical and mechanical properties of coal body. However, the decay rate of impact force in Pingdingshan No. 11 Coal Mine is significantly higher than that in Hengda Coal Mine, and the start time of negative pressure in Pingdingshan No. 11 Coal Mine is significantly earlier than that in Hengda Coal Mine. The start time difference of negative pressure in the two coal mines is about 4000 ms. Due to the different physical and mechanical properties of coal body, the similarity ratio of stress, loading value of ground stress and critical gas pressure are different. It shows that the attenuation of impact force to negative pressure is greatly affected by three factors.
5. According to the damage of impact force to human body^48–50^, the risk division of outburst dynamic disaster is proposed. The impact force greater than 0.0196 MPa is the risk region. By comparing the two coal mines, it is found that the peak value of impact force of Pingmei No. 11 Coal Mine is higher than that of Hengda Coal Mine, but the duration of impact risk is less than that of Hengda Coal Mine. The disaster and damage intensity of Pingmei No. 11 Coal Mine are stronger, and the harm of Hengda Coal Mine is relatively weak. It can be seen that there are commonness and differences in different coal mines, and the specific prevention and control measures should be proposed on the basis of commonness combined with differences.

### Evolution law of gas pressure

Gas pressure is one of the comprehensive effects of outburst, which affects the stages of gestation, development and occurrence of outburst. After the compression molding of coal samples, it is firstly vacuumed for 3 h. When the negative pressure in the cavity is 0.1 MPa, the gas is filled to simulate the adsorption for 24 h. During the test, the gas pressure was gradually loaded from 0.6 to 0.2 MPa every stage. The loading mode selects manual loading. The loading time is generally controlled within 10–20 s, and the voltage stabilization time is 2 min each time. The loading time is relatively short until the outburst occurs.

**Figure 9:**
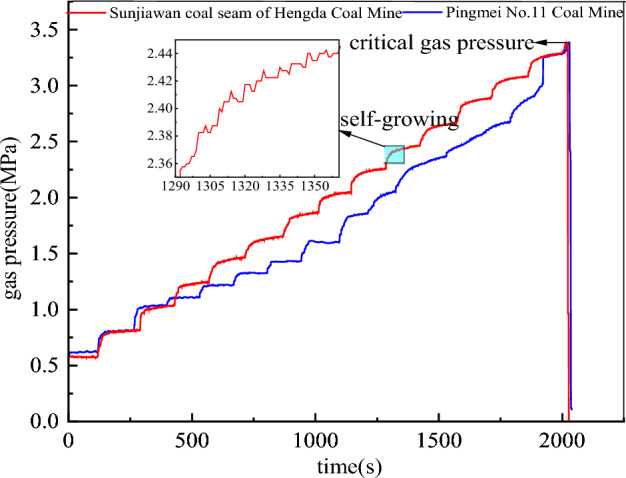
Comparison diagram of changes of the gas pressure.

Before the start of the test, the coal briquette was preloaded to ensure that the coal briquette was saturated and the test chamber was sealed. In the process of pressure stabilization after each stage of loading, new cracks were generated in the coal body. The gas adsorbed on the coal body gradually desorbed and became free, prompting the gas pressure to increase slowly. This increase process is called “self-growing”. Outburst excitation occurs not only in the process of gas pressure loading, but also in the process of gas pressure stabilization. Due to the self-growing characteristics of gas pressure, the briquette specimens continue to fail, and finally meet the outburst conditions, resulting in outburst. Therefore, the adsorption and desorption characteristics of gas are also one of the key factors affecting the occurrence of outburst. According to the actual pre-mining situation, taking the buried depth of 1000 m as an example, the specific changes of gas pressure loading of two coal samples are compared and analyzed, as shown in Fig. 9.

The loading mode of gas pressure of the two coal samples is the same. However, it can be clearly seen from Fig. 9 that the variation curves of initial gas pressure of the two coals basically coincide. As the loading time increases, the variation curves of gas pressure gradually separates. It shows that the moisture content has a certain influence on the characteristics of adsorption of coal on gas^53,54^. That is, water inhibits the adsorption of coal on gas. In the process of specimen making, the moisture content of coal samples in Pingmei No. 11 Coal Mine was raised to 2%, and the moisture content of coal samples in Sunjiawan Coal Seam of Hengda Coal Mine was raised to 5%. At the same time, the coal sample of Sunjiawan coal seam in Hengda Coal Mine has less gas adsorption, more free gas, and higher gas pressure. The coal sample of Pingmei No. 11 Coal Mine has more gas adsorption, less free gas and lower gas pressure. Therefore, the main reason for the separation of the gas pressure change curve with time is that the water inhibits the adsorption of coal on gas. In addition, it is also related to the physical and mechanical properties and structure of coal samples.

**Figure 10:**
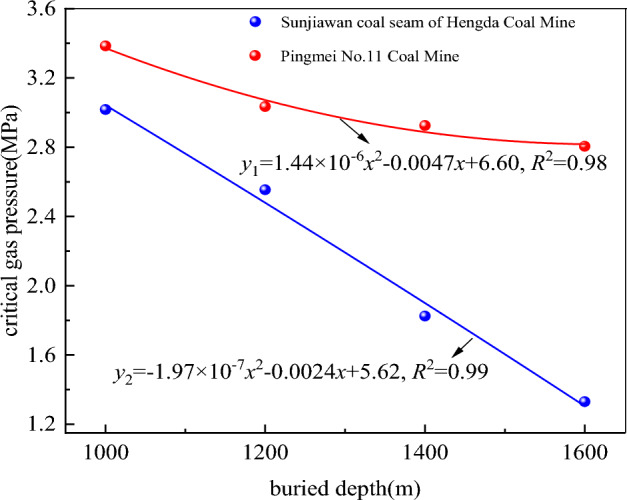
Comparison diagram of the critical gas pressure under different buried depths.

Because it is difficult to collect gas in laboratory test, the instantaneous gas pressure of outburst is defined as critical gas pressure. The influence of buried depth on critical gas pressure is studied. The comparison diagram of the critical gas pressure under different buried depths is shown in Fig. 10. It can be seen from Fig. 10 that the critical gas pressure and buried depth of the two coal samples are in accordance with the nonlinear relationship, and the fitting relationships are:

11 $$y_{1} = 1.44 \times 10^{ - 6} x^{2} - 0.0047x + 6.60(R^{2} = 0.98)$$

12 $$y_{2} = - 1.97 \times 10^{ - 7} x^{2} - 0.0024x + 5.62(R^{2} = 0.99)$$

The curve fitting relationship is good, indicating that the buried depth has a better correlation with the critical gas pressure. The critical gas pressure decreases with the increase of buried depth. This is because the ground stress increases with the increase of buried depth. Under the action of high ground stress, the coal sample is more sensitive to gas pressure, and only a small change in gas pressure will lead to outburst. This is consistent with previous conclusions^55^.

### Influence of gas pressure on the evolution characteristics of impact force

Prominence is the process of energy dissipation. The internal energy of coal before outburst starting is elastic strain energy and gas internal energy. After outburst, energy is mainly converted into coal crushing energy and coal moving energy^56^. The moving energy is provided by the gas internal energy. Part of the gas internal energy is converted into impact kinetic energy, so that the broken pulverized coal and gas air flow are ejected from the pressure chamber, forming a coal–gas impact airflow along the roadway migration and impact, which poses a serious threat to site construction workers and working equipment^30^.

According to the first law of thermodynamics and previous research experience^57,58^, the energy equation of the outburst process can be simplified as :

13 $$W_{1} + W_{2} = A_{1} + A_{2}$$

where $W_{1}$ is the elastic strain energy, ${\text{J}}$. $W_{2}$ is gas expansion energy, ${\text{J}}$. $A_{1}$ is the coal crushing energy, ${\text{J}}$. $A_{2}$ is the coal moving energy, ${\text{J}}$.

The calculation formula of elastic strain energy^59,60^ is:

14 $$W_{1} = {{V\left[ {\left( {\sigma_{H}^{^{\prime}2} + \sigma_{h}^{^{\prime}2} + \sigma_{v}^{^{\prime}2} } \right) - 2\mu \left( {\sigma_{H}{\prime} \sigma_{h}{\prime} + \sigma_{H}{\prime} \sigma_{v}{\prime} + \sigma_{h}{\prime} \sigma_{v}{\prime} } \right)} \right]} \mathord{\left/ {\vphantom {{V\left[ {\left( {\sigma_{H}^{^{\prime}2} + \sigma_{h}^{^{\prime}2} + \sigma_{v}^{^{\prime}2} } \right) - 2\mu \left( {\sigma_{H}^{\prime} \sigma_{h}^{\prime} + \sigma_{H}^{\prime} \sigma_{v}^{\prime} + \sigma_{h}^{\prime} \sigma_{v}^{\prime} } \right)} \right]} {2E}}} \right. \kern-0pt} {2E}}$$

where $V$ is the volume of coal in the outburst range, m^3^. $E$ is the unloading modulus of coal, $E = 326\;{\text{MPa}}$. $\mu$ is Poisson 's ratio. $\sigma_{H}{\prime} ,\;\sigma_{h}{\prime} ,\;\sigma_{v}{\prime}$ are the triaxial stress applied to the coal body test. Through the calculation of Eq. (14), $W_{1} = {39}{\text{.23}}\;{\text{J}}$.

The outburst process is thermally variable due to the influence of ground stress, gas pressure and temperature. In order to be able to quantify the gas expansion energy of the outburst process, it is important to have a clear understanding of the differences in magnitude between the energies involved in the energy transformation process. Considering the short duration of the outburst process, the outburst is treated as an adiabatic process^61,62^, and a simple calculation of the gas expansion energy is carried out, and the free gas expansion energy is calculated by the formula^62–64^ as:

15 $$W_{2}{\prime} = \frac{{10^{3} m_{2} Kp_{1} }}{n - 1}\left[ {\left( {\frac{{p_{2} }}{{p_{1} }}} \right)^{{\frac{n - 1}{n}}} - 1} \right]$$

16 $$K = \frac{1}{{\rho_{p} }} - \frac{1}{{\rho_{t} }}$$

The calculation formula of adsorption gas expansion energy is^64^:

17 $$W_{2}^{^{\prime\prime}} = 41.99m_{2}{\prime} p_{1}{\prime}$$

The calculation formula of gas expansion energy is:

18 $$W_{2}^{{}} = W_{2}{\prime} + W_{2}^{^{\prime\prime}}$$

where $p_{1}$ and $p_{2}$ are the gas pressure of the coal seam before and after the outburst, MPa; $n$ is the gas adiabatic index, taking the value of 1.31; *K* is the porosity of the coal body, %; $m_{2}$ is the overall mass of the coal body, kg; $\rho_{p}$ is the apparent density of the coal type, calculated to be 1.088 (t^.^m^-3^); $\rho_{t}$ is the true density of the coal type, tested to be 1.133 (t^.^m^−3^); the formula for the calculation of adsorption of the expansion energy of the gas is a quantitatively empirical formula, and the value of $m_{2}{\prime}$ and $p_{1}{\prime}$ are taken to be a dimensionless value. is taken as dimensionless value. Calculation can get $W_{2} = 191251\;{\text{J}}$.

The required crushing energy of coal^65,66^ is :

19 $$A_{1} = \sum {\left[ {\left( {{{m_{d} } \mathord{\left/ {\vphantom {{m_{d} } \rho }} \right. \kern-0pt} \rho }} \right)6W\sum {\left( {{{\gamma_{i} } \mathord{\left/ {\vphantom {{\gamma_{i} } {d_{i} }}} \right. \kern-0pt} {d_{i} }}} \right)} } \right]}$$

where $m_{d}$ is the quality of pulverized coal in this area, ${\text{kg}}$. $\rho$ is the density of briquette, ${\text{kg/m}}^{{3}}$. W is the energy consumed to establish a unit new surface area, $W = 0.2\;{\text{J/cm}}^{{2}}$. $\gamma_{i}$ is the proportion of the particle size. $d_{i}$ is the diameter of the particle size, ${\text{m}}$. Through the calculation of Eq. (19), $A_{1} = 112585{\text{J}}$.

The moving work of coal is ^64^:

20 $$A_{2} = \frac{1}{2}m_{1} v^{2}$$

where, $m_{1}$ is to highlight the quality of pulverized coal, kg ; *v* is the instantaneous velocity of pulverized coal, m·s^-1^. Through the calculation of Eq. (20), $A_{2} = 1118\;{\text{J}}$.

The gas expansion energy is 4875 times of the elastic strain energy, which satisfies the previous^57–60^ proposed that the gas expansion energy release is 3–4 energy levels larger than the elastic strain energy release in the outburst process, indicating that the energy in the outburst process mainly comes from the gas internal energy.

The moving work of coal body is 1 118 J, which is 28.5 times of the elastic strain energy, and the gas expansion energy is 171 times of the moving work of coal body. It shows that the elastic strain energy mainly plays a role in the fracture failure of the coal body in the early stage. The coal–gas two-phase flow energy is mainly provided by the gas internal energy, and the gas internal energy determines the strength of the two-phase flow impact force in the roadway.

**Figure 11:**
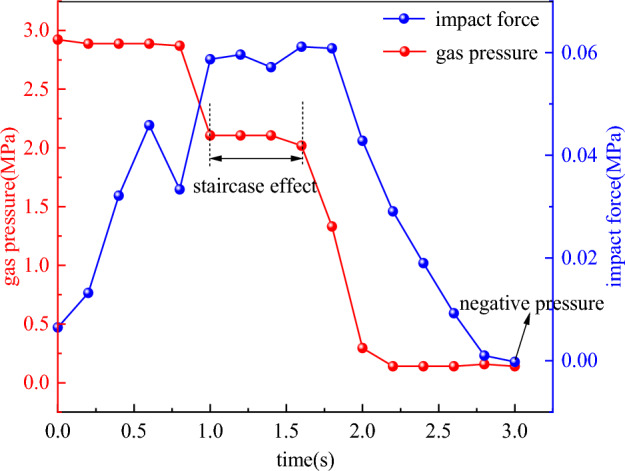
Corresponding relationship between impact force and gas pressure after outburst.

When the gas pressure is loaded to the critical value, the outburst occurs. The impact airflow of coal–gas sprayed into the roadway. The signal is monitored by the impact sensor. Figure 11 shows the corresponding relationship between impact force and gas pressure after outburst, and the following rules are obtained. The change trend of gas pressure is “drop-steady-steep drop-steady”. The change trend of impact force is “steep increase-fluctuation-slow drop-smooth out”.

According to the change trend and energy conversion relationship, the gas internal energy provides energy for the impact force, and part of the gas internal energy is converted into impact kinetic energy, that is, part of the gas pressure is converted into impact force.

The gas pressure begins to decrease when the gas is entrapped by pulverized coal. The impact force increases sharply to the peak. When the channel of two-phase flow is congested, the gas pressure accumulates near the outburst port. The ejection phenomenon occurs intermittently many times. The impact force fluctuates at a higher level. After the gas accumulated in the pressure chamber is gradually ejected, it takes a certain time for the coal body to desorb the gas again. The gas pressure in the cavity decreases and the energy supply is insufficient, so that the impact force begins to decrease slowly. When the coal body no longer desorbs gas, the gas pressure in the pressure chamber approaches atmospheric pressure. The impact force in the roadway tends to be stable after falling, and negative pressure gradually appears.

It can be seen from Fig. 11 that the gas pressure drops sharply after the outburst, but it will not directly drop to atmospheric pressure. It experiences 1–2 times of secondary energy storage process of gas pressure. This process is defined as the staircase effect. Then the gas pressure is close to the atmospheric pressure, indicating that the gas pressure after the outburst has a declining law of stepping, and the gas pressure in the whole outburst process changes step by step.

### The evolution law of impact force and acoustic emission

When the coal body is broken and deformed, the internal concentrated energy is instantaneously released, and the elastic wave phenomenon is called acoustic emission. The research shows that the acoustic emission signal can well characterize the degree of coal fracture and has a corresponding relationship with each stage of the outburst evolution process. Therefore, the correlation analysis of evolution characteristics of impact force and acoustic emission signals can better reflect the degree of coal fracture and its harmfulness.

**Figure 12:**
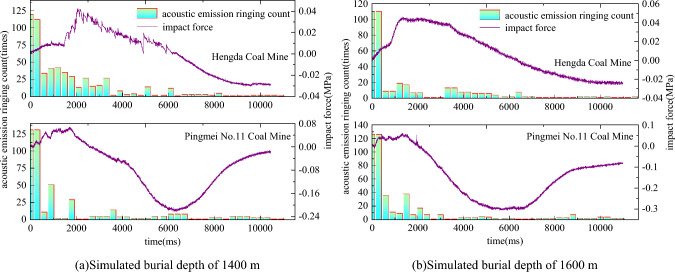
Variation relation between impact force and acoustic emission ringing count.

The relationship between impact force and acoustic emission ringing count after outburst is shown in Fig. 12. The change trend of impact force is “rise-peak-fluctuation of high level-drop”. The change trend of acoustic emission ringing count is “steep drop-slow drop-steady”.

The following regulations are obtained through analysis.

1. The peak point of acoustic emission ringing count is earlier than the impact force. The reason is that the acoustic emission signal monitors the fracture of coal in the cavity and the impact force monitors the migration of two-phase flow in the roadway. After the coal body is broken, it is sprayed out under the gas pressure, forming the impact force of two-phase flow in the roadway.
2. When the impact force fluctuates significantly, the acoustic emission ringing count will also increase accordingly. It shows that the impact force and acoustic emission signal have synchronous monitoring effect. Both of them can reflect the coal fracture after outburst, and realize the mutual verification of various monitoring data. The reliability of the test is confirmed.
3. The change trend of acoustic emission ringing count did not directly drop to the lowest value during the reduction process, and there are many obvious rebounds. The reason is that when the impact force fluctuates, a large amount of energy is accumulated in the coal body, resulting in greater damage. The acoustic emission instrument monitored the occurrence of damage, and the acoustic emission ringing count increased in a small range. Not every impact force fluctuation will be accompanied by a sharp increase in the acoustic emission ringing count. When the outburst occurs to the later stage, the energy accumulation is less, and the impact force fluctuation has less impact. When the impact force of outburst does not cause obvious effect, the acoustic emission signal cannot be monitored and there will be no sharp increase. Therefore, when the acoustic emission ringing count increases sharply, it must be accompanied by the impact force fluctuation, but the impact force fluctuation does not necessarily correspond to the sharp increase of the acoustic emission ringing count.

## Conclusion

1. Through the analysis of the crack propagation mechanism of coal body in the launching area, the propagation mechanism of I-type crack and II-type crack are obtained. With the crack propagation, the generation of I-type crack is a prerequisite for outburst catastrophe. With the crack propagation, the inclination of crack increases, and II-type crack is formed under shear. I-type and II-type cracks intersect and penetrate, resulting in internal structural damage and skeleton instability of coal. Gas wrapped fragmentized coal body thrown, outburst occurs.
2. There is obvious negative pressure in the roadway after outburst. The appearance of impact force attenuation to negative pressure is greatly affected by the physical and mechanical properties of coal body, ground stress and gas pressure.
3. Outburst is the process of energy dissipation. Impact kinetic energy is mainly provided by gas internal energy. Part of the gas pressure is converted into impact force. With the decrease of critical gas pressure, the propagation time of impact force in roadway becomes shorter. It shows that the strength and duration of impact force are determined by the gas pressure. Under deep work conditions, high ground stress and low gas pressure have a better effect on hindering the propagation frequency of impact force in roadway.
4. At the beginning of outburst, the peak point of acoustic emission ringing count is earlier than the impact force. The acoustic emission signal can monitor the outburst hazard earlier. After the beginning of outburst, the impact force and the acoustic emission signal have a synchronous monitoring effect, but the impact force can more specifically reflect the coal fracture. When the acoustic emission ringing count increases sharply, it must be accompanied by the impact force fluctuation, but the impact force fluctuation does not necessarily correspond to the sharp increase of the acoustic emission ringing count.

## Data Availability

The datasets used and/or analysed during the current study available from the corresponding author on reasonable request.
